# Rethinking Sickle Cell Disease as a Systemic Vasculopathy

**DOI:** 10.3390/cells15040326

**Published:** 2026-02-10

**Authors:** Mariana DuPont, Najibah A. Galadanci, Rushil V. Patel, Jeffrey Lebensburger, Julie Kanter

**Affiliations:** 1 Department of Health Sciences and Human Performance, The University of Tampa, Tampa, FL 33606, USA; 2 Division of Hematology and Oncology, Department of Medicine, Heersink School of Medicine, The University of Alabama at Birmingham, 1808 7th Avenue South BDB321, Birmingham, AL 35294, USA; ngaladanci@uabmc.edu (N.A.G.);; 3 Lifespan Comprehensive Sickle Cell Center, The University of Alabama at Birmingham, 1808 7th Avenue South BDB321, Birmingham, AL 35294, USA; 4 Division of Pediatric Hemato-Oncology, Department of Pediatrics, Heersink School of Medicine, The University of Alabama at Birmingham, 1808 7th Avenue South BDB321, Birmingham, AL 35294, USA

**Keywords:** Sickle cell disease, vascular system, multi-organ complications

## Abstract

**Highlights:**

**What are the main findings?**

- Sickle cell disease is a multi-system disorder in which vaso-occlusion, endothelial dysfunction, and chronic inflammation drive progressive vascular-mediated organ damage.
- With increasing age, end-organ complications in sickle cell disease are best understood as manifestations of a systemic vasculopathy in addition to a hematologic disorder.

**What are the implications of the main findings?**

- Advancing outcomes in sickle cell disease will require therapies that address vascular complications in addition to the use of red blood cell-specific therapeutics.
- In sickle cell disease, the development of biomarkers that enable real-time detection of end-organ injury and longitudinal monitoring of organ damage is essential to rigorously assess clinically meaningful outcomes of novel pharmaceutical therapies.

**Abstract:**

Sickle cell disease (SCD) is the most common inherited clinically relevant blood disorder. Although a deceptively simple monogenetic disorder, the associated complications have multiple downstream effects. In this review, we explore the many facets of SCD, with a particular focus on its impact on the vascular system. Despite progress in understanding the underlying mechanisms of SCD, including Hemoglobin S polymerization, microvascular occlusion, and inflammation, there are still many questions surrounding the condition, especially predicting which affected individuals will acquire specific complications in order to personalize treatments. While current standard of care treatments, including hydroxyurea and chronic red blood cell transfusions, have been proven to be disease-modifying, newer therapies like crizanlizumab and voxelotor have only proven to manage symptoms. Newer gene therapies have been approved; however, it is not clear what impact these will have long-term on the end-organ complications of SCD. There is still a significant need to understand how we optimize and personalize therapies to improve outcomes for patients. This review highlights the importance of recognizing SCD as a vascular disease to understand its multi-organ complications and heterogeneity of effects.

## 1. Introduction

Sickle cell disease (SCD) was first identified in 1910 [1,2]. However, despite over one-hundred years of knowledge and discovery, there are still many questions related to outcomes and complications. SCD refers to a group of inherited hemoglobin disorders that render red blood cells RBC more adherent, more fragile, and less able to contain oxygen molecules. When de-oxygenated, the hemoglobin in the RBC polymerizes, resulting in the classic “sickle” shape, leading to the name. Until the 1970s, SCD was mainly a pediatric disease, resulting in death at a median age of 10 years [3] due to infection, stroke, and splenic sequestration. As a result of multiple improvements in early screening and diagnosis, as well as new treatment options, most affected individuals can reach adulthood in high-resource countries. As individuals age, the persistent endothelial dysfunction and anemia result in widespread vasculopathy and end-organ complications in most affected individuals regardless of genotype [4].

The Centers for Disease Control and Prevention (CDC) reports that SCD affects approximately 300,000 births worldwide each year, with at least 100,000 individuals living in the United States [5]. Africa has the greatest frequency worldwide, ranging from 5% to 40% [6], mostly in the sub-Saharan region. In recent years, large populations of affected individuals have also been identified in India and the Middle East, as well as throughout Europe and the United Kingdom. Globally, SCD is the most common, clinically relevant inherited blood disorder affecting approximately 30 million people worldwide.

While originally considered an isolated “blood disorder,” evidence now highlights multiple concomitant pathobiological processes (HbS polymerization, vaso-occlusion, hemolysis-mediated endothelial dysfunction, and inflammation) causing widespread vascular damage and the resulting clinical manifestations of SCD [7]. It is important to understand the vasculopathy caused by SCD to fully appreciate, assess, and treat the breadth of complications. Much of the early understanding of the clinical findings in SCD is the result of the Cooperative Study of Sickle Cell Disease (CSSCD), a large pediatric and young adult registry study. Unfortunately, this study was completed before the widespread implementation of hydroxyurea or the use of prophylactic transfusions for primary stroke prevention and included few people beyond the 5th decade of life [8]. Despite these imperfections, this study remains highly influential in how we consider SCD today, but imparts little information on the current population of aging children and adults who now have the potential to live well into the 7th decade. Further, the CSSCD highlights the complications of homozygous SCD (HbSS), especially in childhood, while downplaying the severity of other types of SCD, which often do not manifest their severity until the 3rd decade of life. More recent data have highlighted these findings and are discussed below.

All types of SCD include the inheritance of HbS. In addition to homozygous SCD (HbSS), which is the most common, there are compound heterozygote manifestations of SCD, including HbSC, and HbSβ-thalassemia and other less common forms, including HbSD and HbSE [9]. As noted, the homozygous condition, HbSS disease, has historically been considered the most severe form of SCD, especially in childhood. However, recent data from several studies have demonstrated that people with other compound heterozygote types of SCD also have significant limitations limiting both quality of life and longevity [4,10]. Often, people with compound heterozygote SCD manifest complications later in childhood, with severity increasing into adulthood.

Pain is one of the most well-known complications of SCD. Acute pain is believed to be caused by acute ischemia, also termed a vaso-occlusive crisis (VOC) or an acute pain crisis (APC). While there are multiple reasons for individuals to experience pain, the VOC or APC is historically defined as an acute painful event lasting at least 2 h in duration for which another etiology cannot be defined. This definition is born from the lack of a laboratory or radiologic test available that can diagnose an area of microangiopathic occlusion. Chronic pain can develop over time due to tissue damage or due to the neurologic effects of repeated pain, as well as more poorly defined mechanisms of acquired hyperalgesia [11]. However, not all affected individuals endure SCD-related pain but still suffer from significant organ dysfunction and early mortality. Vaso-occlusion in the microvasculature is caused by the abnormal adherence of sickled RBCs to the inflamed endothelium, resulting in tissue ischemia. While initially thought to be wholly caused by the hemoglobin polymerization and RBC sickling, extensive data have demonstrated that vaso-occlusion can be initiated by white blood cells or reticulocytes, especially in areas of previous endothelial damage [12]. Importantly, vaso-occlusion occurs in affected individuals and does not always result in the acute pain or VOC, thereby sometimes underestimating disease burden.

Although hemoglobin polymerization remains the root cause of SCD, there are multiple heterogeneities that remain poorly understood. Previous researchers have discerned notable patterns within the SCD phenotype, often divided into a “vaso-occlusive” phenotype or a “hemolytic” phenotype, but this classification oversimplifies the complexity of SCD [13]. In actuality, many individuals display elements of both hemolysis and vaso-occlusion, while others remain free of any significant organ damage or pain-related complications. Due to a scientific failure to maintain sufficient longitudinal clinical registries to better elucidate clinical phenotypes, the biological understanding of SCD currently exceeds the clinical knowledge. Here, we focus on viewing SCD as a clinical vascular disease, identifying both current and future therapeutic targets, and emphasizing the importance of developing an improved clinical predictive capacity.

## 2. Pathobiological Mechanisms of Vascular Damage

There are four main pathobiological mechanisms underlying clinical illness in SCD, including HbS polymerization, vaso-occlusion, endothelial dysfunction, and sterile inflammation. Each mechanism contributes to vascular damage and explains the complications associated with SCD.

### 2.1. HbS Polymerization

Polymerization is a chemical process where small molecules known as monomers combine to form a larger, chain-like, or networked molecule called a polymer. The pathophysiology of SCD involves defective HbS polymerizing following deoxygenation in the tissues, causing RBCs to form fibers that distort and obstruct blood flow [7,14,15]. In addition, HbS polymerizes and disrupts the regular structure of the lipid bilayer and proteins in RBC membranes. This disruption leads to a decrease in cellular hydration and an increase in hemolysis, which results in the death of RBCs. The polymerization also changes the structural architecture of the RBC, causing increased adherence of the cellular membrane.

### 2.2. Vaso-Occlusion

In addition to pain, vaso-occlusion in people with SCD results in significant organ damage, such as acute chest syndrome (sickling and adhesion within the pulmonary vasculature resulting in limited oxygen exchange), ischemic stroke due to micro- or macro vessel blockage in the brain, bone damage, and other types of organ failure. Vaso-occlusion causes hypoxia, ischemia, and tissue injury, followed by persistent vascular inflammation, which underpins many aspects of pain and damage in SCD [16,17]. The acute VOC is caused by the combination of tissue ischemia and reperfusion injury, both of which result in nociceptive pain [18]. Importantly, the instigator of vaso-occlusion in many situations is unknown. While data have shown that inflammation, stress, increased viscosity, reduced flow, hemolysis, hypoxia, dehydration, acidosis, or can initiate the vaso-occlusive cascade, there are other times in which no identifying etiology can be determined [7,19]. 

### 2.3. Endothelial Dysfunction

Vascular endothelial dysfunction results from hemolysis and is caused by the direct damage of vaso-occlusion [20]. Hemolysis releases cell-free hemoglobin, which generates severe oxidative stress, causing impairment of endothelial function. Simultaneously, intravascular hemolysis and resultant release of free hemoglobin potentiates the scavenging of NO, leading to regional deficiency of NO [21]. This NO depletion changes the homeostatic vascular processes such as vasodilation, suppression of platelet activation, inhibition of endothelial adhesion molecule, endothelin-1 production, and regulation of intimal and smooth muscle proliferation. As hemolysis increases, patients become more prone to vascular injury and organ dysfunction. The endothelial dysfunction then causes upregulation of key adhesion molecules, including selectins (P- and E-) and vascular cell adhesion molecule-1 (VCAM-1), resulting in red and white blood cell adhesion [7,22].

### 2.4. Inflammation

**Figure 1:**
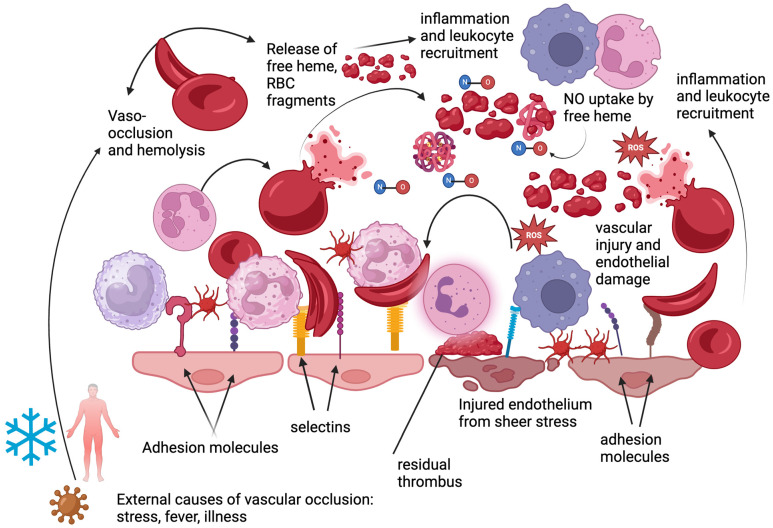
The Vicious Cycle of Inflammation in Sickle Cell Disease.

Hemolysis results in the release of free heme, which significantly contributes to persistent inflammation in individuals with SCD. Independently, free heme leads to activation of TLR4 and inflammatory pathways. Activation of TLR4 on monocytes and other innate immune cells leads to downstream nuclear factor κB (NF-κB) signaling and the release of pro-inflammatory cytokines and chemokines, including tumor necrosis factor–α (TNF-α), interleukin (IL)-1β, and IL-6. These cytokines amplify endothelial activation by increasing expression of adhesion molecules, promoting leukocyte recruitment, and impairing endothelial nitric oxide signaling. In SCD, sustained elevation of these inflammatory mediators contributes to endothelial dysfunction, increased vascular permeability, and propagation of vaso-occlusive events. In combination with repeated vaso-occlusion and reperfusion events, the release of free heme results in ROS production, microvascular dysfunction, and activation of innate and adaptive immunological responses [23,24,25]. As inflammation develops and chemokines recruit additional leukocytes and platelets, the likelihood of worsening vaso-occlusion increases, resulting in a vicious cycle as shown in Figure 1.

### 2.5. Systemic Vasculopathy

Although a disease of RBCs at the genetic level, vascular complications are the major cause of the significant morbidity and mortality seen in SCD. Similar to diabetes, the vasculopathy is caused by direct trauma to the endothelium and by metabolic and hemodynamic factors in SCD. Vascular damage occurs at both the micro- and macro-vascular level, resulting from the combination of sheer stress, chronic nitric oxide (NO) deficiency, and inflammatory damage. Previous studies focusing on endothelial dysfunction in SCD have explained how NO scavenging by cell-free plasma hemoglobin results in decreased bioavailability of endogenous NO, leading to chronic NO deficiency. These studies highlight how the chronic NO deficiency then causes vasoconstriction, proliferative vasculopathy, pulmonary hypertension, and activation of platelets [26,27,28]. While NO deficiency certainly contributes to the vascular damage in SCD, individuals with variants of SCD with less hemolysis (HbSC disease, as an example) also exhibit significant vascular damage, suggesting that the vascular injury is multi-factorial. In this scenario, it can be hypothesized that both the rigidity of the hemoglobin within the RBC and the adhesiveness of the RBC membrane (in combination with those people with SCD who have higher total hemoglobin) result in higher viscosity and subsequent increased sheer stress damage to the endothelium. The combination of the abnormal NO metabolism with the direct trauma to the endothelium can account for the vascular stenosis seen in non-proliferative types of SCD. Similarly, hypothetical, people with SCD who have lower hemoglobin and vascular compromise likely have more distal ischemia, causing neo angiogenesis resulting in a proliferative vasculopathy.

## 3. Current Approved Treatments for SCD and Their Effect on Vasculopathy

As explained, the pathophysiology of SCD is caused by four overlapping pathophysiologic processes, including (a) hemoglobin S polymerization, (b) increased adhesion-mediated vaso-occlusion, (c) hemolysis-mediated endothelial dysfunction, and (d) activation of inflammation. Now that there is a better understanding of the mechanisms of cellular injury, these pathways are also the targets for therapeutics in SCD.

There are currently four disease-modifying drugs for SCD approved by the US Food and Drug Administration. These include Hydroxyurea, L-glutamine, crizanlizumab, and voxelotor [29,30,31,32]. Additionally, new genetic therapies, Exagamglogene autotemcel and Lovotibeglogene autotemcel, were also recently approved by the FDA [33]. To best understand the efficacy of these therapies, we look at how each treatment specifically affects SCD-related pathophysiology.

### 3.1. Hydroxyurea

Hydroxyurea has several mechanisms of action in SCD. First, it is an inhibitor of ribonucleotide reductase that induces fetal hemoglobin (HbF), although the mechanism of HbF induction remains unclear [34,35,36]. HbF prevents HbS polymerization in SCD, decreasing both vaso-occlusion and reducing hemolysis. Hydroxyurea also increases available nitric oxide, which reduces red cell adhesion [37]. Finally, hydroxyurea also suppresses bone marrow production, decreasing both leukocyte and reticulocyte production, which results in decreased overall inflammation, also reducing RBC adhesion and vaso-occlusion [38,39].

Hydroxyurea was initially approved for adults with SCD by the US FDA in 1998 based on the randomized controlled trial that demonstrated decreased incidence of acute pain episodes and ACS with hydroxyurea [29]. In 2014, the National Heart, Lung, and Blood Institute (NHLBI) expert panel strongly recommended the use of hydroxyurea to all individuals with SCD above 9 months of age [40]. Since that time, hydroxyurea has become the standard of care for reducing the risk of vaso-occlusive episodes, anemia, hemolysis, and chronic organ dysfunction in children and adults with SCD. More recently, hydroxyurea has also been demonstrated to be effective for stroke prevention in areas in which chronic red cell transfusion is inaccessible [41,42]. Similarly, the TWITCH trial also demonstrated that hydroxyurea could be used to reduce the rate of abnormal transcranial Doppler (TCD) or sickle stroke screen for children with SCD. These notable effects of hydroxyurea validate that it can curb or delay the development of vasculopathy in people with SCD.

### 3.2. L-Glutamine

Pharmaceutical-grade L-glutamine, approved by the US FDA in 2017, is a conditionally essential amino acid and a precursor of nicotinamide adenine dinucleotide (NAD) [43]. L-glutamine has a complex indirect mechanism of action in SCD that improves NAD redox potential, thereby leading to decreased oxidative stress of red blood cells, improved red blood cell health, decreased endothelial adhesion, and ultimately decreased number of pain crises [44]. L-glutamine was shown to decrease the number of pain crises, frequency of acute chest syndromes, and hospitalizations in individuals with SCD [30]. While demonstrated to reduce inflammation in vitro, the long-term effect on vascular damage or organ function in SCD has not been shown.

### 3.3. Crizanlizumab

Approved by the FDA in 2019, crizanlizumab is a humanized monoclonal anti-P-selectin antibody that blocks the interaction between P-selectin, which is expressed on endothelial cells and platelets, and P-selectin glycoprotein ligand 1 [31]. This leads to decreased adhesion between the endothelium and endothelial cells, platelets, sickled red blood cells, and leucocytes, thereby decreasing inflammation. Crizanlizumab is currently approved only in the United States for the reduction of vaso-occlusive complications in patients ≥ 16 years of age. While conceivable that crizanlizumab should prevent the development of ongoing vasculopathy in SCD, there is currently no definitive evidence demonstrating long-term effect.

### 3.4. Voxelotor

Voxelotor was also approved by the US FDA in 2019 [45] for treating patients with SCD ≥ 12 years of age [46]. Voxelotor prevents HbS polymerization by forming a reversible covalent bond with the N-terminal valine of the α chain of hemoglobin [47]. This bond changes the conformation of hemoglobin, thereby increasing Hb oxygen affinity, leading to decreased deoxygenated HbS, decreased RBC sickling, improved deformability, leading to reduced hemolysis and increased Hb [32]. Clinical trials have demonstrated that voxelotor improves hemoglobin by a minimum of 1 g/dL and significantly decreases hemolysis. Currently, however, post-marketing safety concerns have emerged, prompting regulatory actions. In the US, safety warnings have been issued [48,49], and in Europe, voxeletor use has been suspended by regulatory authorities. As a result, voxelotor was voluntarily withdrawn from the global market by the manufacturer, Pfizer, in September 2024. In addition to the noted safety concerns, the medication has not been shown to conclusively reduce acute pain crises or to reduce the incidence of organ damage in SCD. Theoretically, the early initiation of this medication should decrease the prevalence of free heme release, allowing for less inflammation and vascular damage; however, there is no clear evidence of this long-term effect at this time.

### 3.5. Red Blood Cell Transfusion

One of the mainstays of treatment for severe acute and chronic SCD complications has been RBC transfusion. Over 90% of adults with SCD have received at least one transfusion in their lifetimes [50]. The goal of RBC transfusion in people with SCD includes increasing oxygen carrying capacity, decreasing risk of SCD complications, and suppressing endogenous HbS-laden RBC production [51]. Chronic transfusions using monthly simple or exchange transfusions help prevent long-term complications by replacing the rigid erythrocytes with normal deformable cells and suppressing the formation of sickle erythrocytes [52]. Long-term RBC transfusion therapy is commonly given to patients with SCD for the management of both acute and chronic complications [52]. RBC exchange is now the standard common practice for treating SCD-related complications, where RBCs from a healthy matched donor are infused while the patient’s RBCs are simultaneously removed. The advantages of RBC exchange over simple RBC transfusion are the increase in oxygen carrying capacity without increasing viscosity, while removing cells susceptible to sickling, thereby decreasing the likelihood of vaso-occlusive crisis (VOCs).

Children with SCD have a 100-fold increased risk of developing stroke compared to children without SCD, with 70% of these children developing recurrent strokes in the absence of secondary stroke prevention [53]. Anemia is the most common cause of ischemic stroke in these children and is the most common indication for chronic transfusion. The role of anemia in ischemic stroke has been confirmed by the landmark clinical trial showing that chronic prophylactic transfusions prevent stroke in 90% of children with high cerebral blood flow velocities as measured by transcranial Doppler ultrasound scan [54]. The primary goal of transfusion as a treatment for acute stroke is to reduce HbS and rapidly reverse or stop the progression of the brain vasculature occlusion. Further, chronic transfusion treatment with appropriate HbS suppression helps prevent secondary strokes, and discontinuation has been shown to be associated with recurrence.

While red blood cell transfusion remains a cornerstone of SCD management, it is associated with several well-recognized risks. Individuals with SCD are at increased risk of red cell alloimmunization, delayed hemolytic transfusion reactions, and hyperhemolysis, complications that can be life-threatening and are more common in this population due to repeated transfusions and donor–recipient antigen mismatch. In addition, transfusion-related infections and iron overload remain important concerns, particularly in regions with limited access to extended antigen matching, leukoreduction, and chelation therapy. These risks underscore the importance of careful patient selection, optimal transfusion practices, and the need for safer alternatives to chronic transfusion therapy.

### 3.6. Allogeneic Hematopoietic Stem Cell Transplant

Allogeneic hematopoietic stem cell transplantation (HSCT) is the only established curative treatment for SCD [55]. Allogeneic HSCT leads to complete or partial donor-derived normal erythropoiesis, resulting in cure. Additionally, HSCT has been shown to stabilize and restore function in affected organs, including the CNS and lung, and prevent further deterioration of function. Several studies performed in France have demonstrated that individuals with SCD with abnormal TCD (sickle stroke screen) or with a history of overt stroke who have undergone HSCT with a matched related donor do not have recurrence of these abnormalities [56]. These findings suggest that endothelial and vascular remodeling may be possible when there is persistence of normal erythropoiesis without ongoing hemolysis and vascular damage. Interestingly, there is little data on the effect of HSCT on other organ systems, including the heart, kidneys, eyes, or penis, all organ systems discussed below as emblematic of the vascular damage in SCD. While multiple articles demonstrate successful outcomes of HSCT in SCD, specific research is needed to understand the long-term effects on vascular damage after transplant and whether vasculopathy can be both prevented or altered.

### 3.7. Genetic Therapies

The current approved mechanisms of gene therapy include one gene addition-mediated treatment and one using gene editing [57]. In addition, a new therapeutic gene is inserted into the original intact genome, using a viral vector, leading to the synthesis of a new non-sickling hemoglobin molecule. The current gene added is the anti-sickling β globin gene, which is a mutated version of the β globin gene that has additional anti-sickling activities. Data from the original trials of Lovotibeglogene autotemcel (lovo-cel) have demonstrated significant improvement in hemoglobin and reduction of hemolysis, as well as reduction in acute pain crisis and improvement in patient-reported outcomes [58]. The second approved mechanism is the use of gene editing to enhance HbF expression. As noted above, fetal hemoglobin is a naturally occurring anti-sickling hemoglobin and (when paired with HbS) results in decreased hemolysis and improved oxygen delivery. The current therapy, Exagamglogene autotemcel (Exa-cel), targets a transcription factor (BC11a) away from the HbF gene. Using a CRISPR-CAS9 technology to create a double-stranded DNA break in the BC11a gene, it can reverse the normal suppression of HbF production caused by this protein, leading to increased HbF induction while reciprocally suppressing HbS production. Similar to lovo-cel, the exa-cel therapy has demonstrated a significant biologic improvement in affected persons with higher hemoglobin, remarkably decreased hemolysis, and reduction of vaso-occlusive pain events [59]. At this time, it is too early to assess whether these therapies can reduce or remove the threat of vascular complications from affected persons. Currently, both therapies result in a transformative biologic effect with near normalization of hemolysis, improvement in hemoglobin, and a significant reduction in clinical pain events. However, it is too early to assess whether these findings will translate into stabilized organ dysfunction, prevention of further organ dysfunction, or improvement in longevity.

### 3.8. Emerging Inflammation Targeted Therapies

Increasing recognition of inflammation as a central driver of vaso-occlusion, endothelial dysfunction, and organ injury in SCD has led to the development of novel therapeutic strategies aimed at directly modulating the inflammatory axis. Recombinant ADAMTS13 (rADAMTS13) is currently under clinical evaluation to address the relative functional deficiency of ADAMTS13 observed in SCD, which contributes to endothelial activation and microvascular thrombosis [60]. Complement pathway dysregulation has also been implicated in SCD pathophysiology, and crovalimab, a monoclonal antibody targeting complement component C5, is being investigated for its potential to reduce complement-mediated inflammation and vascular injury [61,62]. Additional approaches include targeted inhibition of pro-inflammatory cytokines, such as IL-1β, IL-6, and TNF-α, which are known to contribute to endothelial dysfunction, leukocyte activation, and vaso-occlusion in SCD. Although these agents are not yet approved for SCD, early-phase studies suggest potential benefit in carefully selected populations [63]. Multi-target strategies, including omega-3 fatty acid supplementation, are also being explored due to their anti-inflammatory, antithrombotic, and endothelial-protective properties [64,65]. Collectively, these emerging therapies highlight a shift toward interventions that directly address inflammation and vascular injury in SCD and may complement existing disease-modifying treatments.

## 4. Vasculopathy in Sickle Cell Disease: Organ by Organ

### 4.1. Stroke

A clinical (or overt) stroke is defined as an abnormal magnetic resonance imaging (MRI) of the brain with a localized neurological impairment that lasts for an extended period of time, normally more than 24 h [66]. A silent stroke is defined as an abnormal MRI of the brain with a normal neurologic examination. For individuals with SCD, the lifetime risk for an overt stroke is 25% to 30%, historically greatest during the early ages of life (historical data have shown that between 5% and 17% of SCD patients can experience their first stroke between infancy and adolescence) [66]. Almost one-third of adolescent/young adult patients will develop a silent stroke, and the prevalence increases to almost 50% of adult patients with sickle cell anemia [67]. Clinical or overt strokes can be categorized as either an ischemic or a hemorrhagic stroke. Historically, the majority of strokes in people with SCD have been ischemic strokes [68,69], especially in early childhood. The increased risk of stroke in SCD is due to both a progressive vasculopathy resulting in narrowing of key cerebral blood vessels and impaired oxygen delivery due to HbS-laden RBCs in areas of the brain that require increased metabolic demand. However, as people age into adulthood, the percentage of hemorrhagic strokes increases, likely due to a combination of worsening cerebral vasculopathy, including the development of moya moya or aneurysms [70,71].

Moya moya disease is the development of collateral vessel formation due to a progressive narrowing of the intracranial internal carotid arteries, low oxygen delivery, and the resultant development of angiogenesis [72]. Unfortunately, the newly formed vasculature is prone to rupture, thereby increasing the risk of hemorrhagic stroke [73,74]. There are no established screening protocols for moya moya (guidelines recommend MRI screening but not MRA), so the prevalence of moya moya in SCD patients remains unknown and may be underestimated [75,76].

An aneurysm is an unusual ballooning in the wall of a blood vessel, usually an artery. Both pediatric and adult patients with SCD can develop aneurysms, likely due to ongoing vascular damage and repair weakening the vessel wall. These aneurysms may occur in atypical locations (from people without SCD) in both the anterior and posterior circulations [77,78]. Further, aneurysm development in people with SCD does not correlate with typical risk factors like hypertension, renal disease, cigarette smoking, or connective tissue disorders. Instead, their pathogenesis is linked to hypoxia, sickling, and endothelial injury [79]. With HbSS, intracranial aneurysms are frequently seen, particularly among women aged 30 to 39 years [78]. However, without formal screening protocols, the true prevalence of these vascular disruptions is not known. It is clear that adults with SCD have a higher chance of developing aneurysms [77].

The use of TCD ultrasound, also term “the sickle stroke screen,” was an essential discovery in the prevention of ischemic stroke in children with SCD. In the landmark stroke prevention trial, patients with an abnormal (high velocity) TCD who were randomized to chronic red cell transfusions (CRCT), as compared to standard care, demonstrated a 92% reduction in stroke risk [54]. In clinical settings in which CRCT is feasible, all patients with an abnormal TCD should be initiated on transfusion therapy to reduce the risk of stroke (primary stroke prevention). Subsequently, a randomized trial of hydroxyurea demonstrated that patients who had been started on CRCT for an abnormal TCD but who did not have abnormal vasculature on magnetic resonance angiography (MRA) could be transitioned to hydroxyurea for continued primary stroke prevention [80]. This same study highlighted the importance of continuing CRCT for those individuals who did have significant intracranial vasculopathy. Other newer studies have also shown that CRCT and hydroxyurea have recently been shown to improve oxygenation of the brain and reduce the need for increased cerebral blood flow [81,82,83]. Further, hydroxyurea may be effective in reducing the risk of patients developing either an abnormal “sickle stroke screens”/TCD [84] or developing an overt stroke [41,85].

Importantly, for those children or adults who have experienced a clinical (overt) stroke, secondary stroke prevention with CRCT is key, as up to 2/3 of patients will have a recurrent stroke if left untreated [53]. CRCT remains the treatment of choice with a goal of maintaining a consistent level of HbS < 30% and using optimally matched RBC [86].

### 4.2. Cardiac Complications of SCD

As explained above, SCD is characterized by recurrent episodes of ischemic injury, vascular damage, remodeling, and hemolytic anemia resulting in progressive damage to vital organ systems, including the heart [87]. Importantly, the heart has previously been theorized to be more protected from the effects of SCD as it is perfused with highly oxygenated blood that should limit the actual polymerization of HbS. However, the increasing frequency of cardiac complications in adults with SCD (compared to children) demonstrates that much of the damage induced is the result of ongoing vascular injury from repeated adhesion/inflammation and not solely from hemolysis and sickling [88]. As individuals with SCD age, however, they are at an increased risk for developing various cardiac complications.

As with other elements of SCD, there are multiple, overlapping, and synergistic causes of cardiac damage in SCD. Understanding and preventing the development of SCD-induced cardiomyopathy is paramount, given that recent publications note that cardiac failure is a frequent cause of death in the adult population [89]. The cardiomyopathy of SCD includes the progressive proliferation of systemic vasculopathy, left ventricular diastolic dysfunction, ventricular hypertrophy, atrial dilation, and pulmonary hypertension (PH). These changes can have significant implications for the long-term health and well-being of individuals with SCD, and as such, research efforts are underway to better understand the underlying mechanisms and identify effective treatment strategies [87]. Pulmonary hypertension has been discussed as one of the most significant biomarkers of mortality in SCD and will be explored further below.

Chronic anemia in SCD also contributes to the pathological development of cardiomyopathy in SCD. Anemia results in dilation of the atrial chamber and a compensatory increase in left ventricular mass, resulting in left ventricular hypertrophy that can also be associated with left ventricular diastolic dysfunction. Data from recent studies have shown that cardiac damage can start early in childhood, although its progressive nature is poorly understood [90]. Studies have also shown that diastolic dysfunction is associated with marked abnormalities in exercise capacity in these patients and may also predict mortality [91].

Early in childhood, the chronic anemia associated with SCD results in an increase in cardiac output. Left ventricular stroke volume then increases, resulting in significant hypertrophy and dilation of the left ventricle [92]. The diastolic abnormalities increase with older age, simultaneous with an increase in blood pressure, increased LV mass (hypertrophy), and the development of renal dysfunction. While many people with SCD-induced cardiomyopathy also have hypertension, the link to worsened cardiac disease is not clear due to the combination of compensatory hypertrophy secondary to anemia and LV dilation, along with a systemic vasculopathy affecting afterload [93].

Other likely contributors to cardiac abnormalities in SCD are the direct myocardial damage from microvascular disease and inflammation. A recent study suggested that widespread myocardial fibrosis diagnosed using cardiac MRI may be present and also contributing to the diastolic dysfunction described above [94]. Interestingly, despite the significant damage to the heart in SCD, multiple studies have reported infrequent events of classical myocardial ischemia or infarction. Classical myocardial ischemia is diagnosed using electrocardiographic (ST elevation and ST and T wave changes) combined with elevated circulating biomarkers of myocardial injury [95]. SCD can cause myocardial ischemia due to poor oxygen delivery and nitric oxide deficiency, resulting in abnormal endothelial and smooth muscle dysfunction. This type of ischemia may be more subtle and not diagnosed using electrocardiography. Further, the cardiac ischemia in SCD may be quickly reversed by reperfusion, which can manifest differently than a classical myocardial infarction, though the reperfusion can also lead to further injury due to oxidative stress with a more chronic, protracted etiology. Further, evidence supports that the use of serum cardiac enzymes to diagnose myocardial injury in SCD may not be as reliable in SCD due to hemolysis and skeletal muscle injury, highlighting the need for other diagnostic biomarkers in this population [96]. Clearly, the etiology of cardiovascular damage caused by vascular abnormalities is different in SCD, as the formation of coronary atherosclerosis with cholesterol plaque formation seen in other MI patients rarely occurs in SCD [97]. However, cardiac damage and subsequent cardiac death still occur.

### 4.3. Pulmonary Hypertension

Pulmonary hypertension (PH) is another common vascular complication in adults with SCD and is defined as a mean pulmonary artery pressure (mPAP) of 25 mm Hg combined with a mean pulmonary artery wedge pressure (PAWP) of 15 mm Hg. Classical PH is classified into five major clinical categories that focus on the underlying cause of abnormal pulmonary artery pressure: pulmonary arterial hypertension (PAH; group 1); left heart disease (group 2); lung diseases and/or hypoxia (group 3); pulmonary artery obstructions (particularly thromboembolic syndromes) (group 4); and undifferentiated or multifactorial causes (group 5), which include SCD and sarcoidosis [98,99]. Most individuals with SCD are categorized as group 5 due to the multifactorial vascular pathophysiology that may include vascular injury in addition to other cardiac abnormalities due to severe anemia. Some individuals with SCD may exhibit more classic PH characteristics associated with left-sided cardiac disease (group 2) or thromboembolic illness (group 4). The exact pathogenesis of PH in SCD is not yet fully understood, but it has been linked to repeated endothelial damage, NO depletion, hypercoagulability, and acute and chronic inflammation [100,101,102,103,104,105,106]. It has been reported that PH affects 6 to 11 percent of people with SCD, with less than half of them having precapillary PH [107,108].

**Figure 2:**
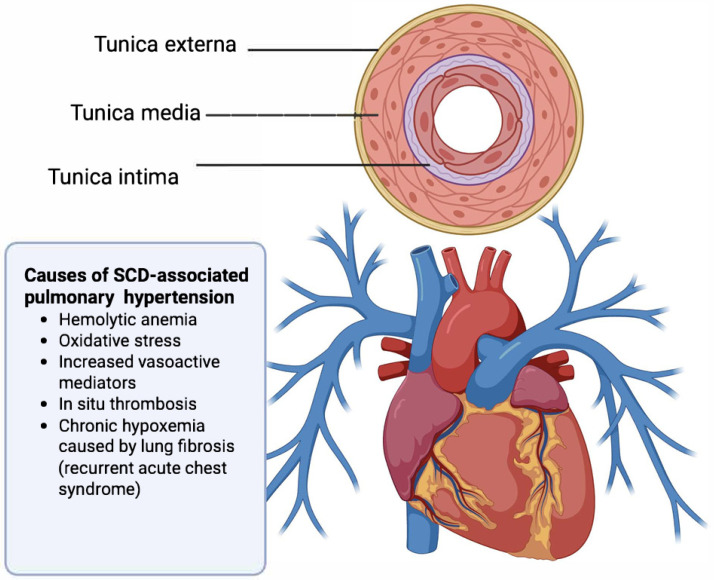
Mechanisms of pulmonary hypertension in people with SCD. A pulmonary vessel with smooth muscle proliferation is shown, representing a person with SCD with pulmonary arterial hypertension. Mechanisms contributing to the development of PH are summarized. Adapted from Gladwin et al. 2012 [87].

In epidemiological studies, the development of PH is associated with hemolysis/endothelial dysfunction, leg ulceration, kidney disease, iron overload, and liver dysfunction, though its exact pathogenesis remains unclear (Figure 2) [87]. The diagnosis of pulmonary hypertension currently requires patients to undergo a right heart catheterization. However, the risks and costs associated with a right heart catheterization do not make it an ideal screening test. While echocardiogram findings can suggest a risk for mortality, the use of screening echocardiography to diagnose PH in SCD remains controversial. The American Society of Hematology recommends screening in people with SCD with cardiopulmonary symptoms (hypoxia, dyspnea, shortness of breath, etc.) while the American Thoracic Society recommends annual screening of every adult living with SCD [109]. Clearly, more research is required to improve the identification of which individuals need echocardiogram screening.

There are no clear treatments for cardiomyopathy, including PH in people with SCD [50]. However, both hydroxyurea and CRCT have shown efficacy in small studies [110]. At present, hydroxyurea is the preferred initial treatment for people with SCD who develop PH [109]. However, the therapeutic benefits of hydroxyurea may not become apparent until several months after initiation. Thus, as per the American Thoracic Society, patients who are unresponsive to hydroxyurea may benefit from other treatments such as CRCT.

Mechanistically, CRCT using erythrocytapheresis should improve and possibly prevent cardiopulmonary dysfunction or damage by improving oxygen delivery and reducing cardiac stress. Additionally, less vaso-occlusion should result in less cardiac tissue damage and fibrosis, which should also reduce cardiac damage. However, this assumption is not yet proven either as an individual treatment or in combination with other therapies such as hydroxyurea. Sildenafil, a phosphodiesterase inhibitor, has been shown to improve exercise capacity in patients without SCD with pulmonary arterial hypertension. Unfortunately, results from the recent study, Treatment of Pulmonary Hypertension and Sickle Cell Disease With Sildenafil Therapy (walk-PHaSST study), showed that sildenafil did not have a similar effect in patients with SCD with increased TRV and low exercise capacity (based on a six-minute walk distance). In this study, there was no evidence of a treatment effect on the six-minute walk distance, TRV, or N-terminal pro-brain natriuretic peptide. Further, the trial was stopped early due to the finding that sildenafil leads to increased hospitalization for pain in patients with SCD [28,111]. The same team is now conducting a large study funded by the National Institute of Health to assess whether the initiation of CRCT in these high-risk individuals with SCD may result in improvement in cardiopulmonary function [112].

### 4.4. Priapism

Priapism is a medical condition characterized by a prolonged, unwanted erection that persists for more than four hours. Priapism is more commonly observed in males, though rare occurrences of clitoral priapism in women with SCD have also been reported [113]. Priapism is considered a medical emergency; within six hours of the onset of priapism, the penis undergoes physiological changes and tissue damage. In some cases, permanent structural changes can occur by 12 h, caused by swelling between the trabecular fibers of the corporal smooth muscle tissue, resulting in cellular damage leading to thinning and weakening of the basement membrane and subsequent tissue necrosis [114,115]. Priapism can result in long-term tissue damage and requires immediate assessment by a urologist and often requires manual decompression [116,117].

**Figure 3:**
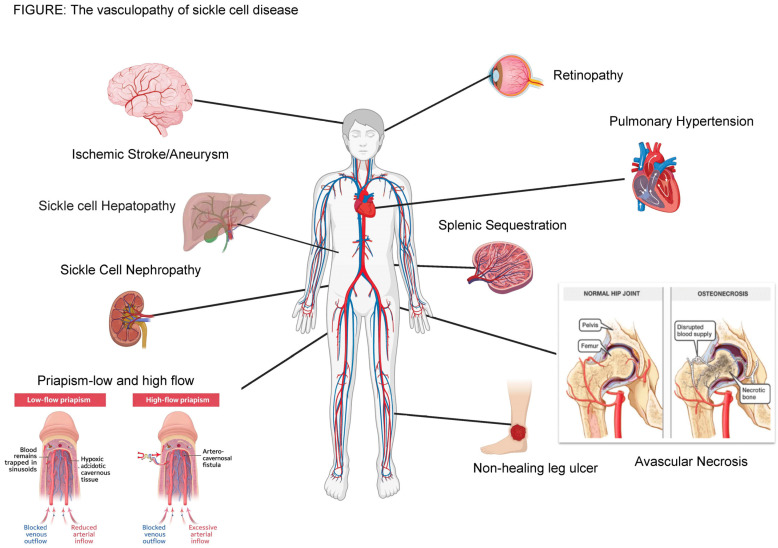
The Vasculopathy of Sickle Cell Disease.

Priapism is categorized as either low arterial inflow into the corpora cavernosa (ischemic), unregulated arterial blood flow into the corpora cavernosa (nonischemic/arterial), or recurrent (stuttering). During a normal erection, phosphodiesterase deactivates cyclic GMP, which causes smooth muscle contraction [118]. Nitric oxide also plays a crucial role in normal erectile function, and medications like sildenafil and other phosphodiesterase type 5 inhibitors can prevent the breakdown of cyclic guanosine monophosphate, leading to improved and extended intracavernosal smooth muscle relaxation [118]. However, in individuals with SCD, normal erectile function may be flawed due to previously described endothelial dysfunction and vaso-occlusion. Ischemic priapism is the most common type of priapism in individuals with SCD; studies estimate its occurrence in 33% of affected males [119], as shown in Figure 3.

To date, there is no effective SCD-modifying drug proven to treat priapism. Low-dose PDE5 inhibitors, including sildenafil, when taken daily, have demonstrated some benefit in reducing ischemic priapism recurrence [120,121,122]. The rationale for therapy is to restore NO balance with improved PDE5 function. Although this shows potential for patients with recurrent priapism, this should be used with caution due to the increased risk of VOC crises and hospitalization for SCD patients treated with sildenafil in the walk-PHaSST trial [111]. Of note is that the dose and frequency used for the walk-PHaSST trial are significantly higher than recommended for priapism. More recent results from ongoing trials of crizanlizumab also show some promise. The SPARTAN trial is a phase 2 trial to assess the efficacy and safety of crizanlizumab in SCD patients with priapism. In the trial, patients aged ≥ 12 years with any SCD genotype and ≥4 priapic episodes lasting ≥60 min over 14 weeks received crizanlizumab. Results of the interim analysis show that crizanlizumab treatment over 26 weeks reduced priapism events by approximately half compared to baseline [123].

### 4.5. Leg Ulcers

Non-healing leg ulcers are the most common skin manifestation among individuals with SCD. Global factors implicated in the development of leg ulcers in SCD include an initiating injury to the skin, poor perfusion, edema arising from either venous stasis and/or anatomic dysfunction, hypoxia, decreased bioavailability of nitric oxide, and microvascular thrombosis [124]. While these contributing factors have been delineated, the pathophysiology remains poorly understood and likely heterogeneous. Excessive RBC adhesion and sickling prohibit tissue perfusion while increasing coagulability and leading to recurrent pain. Then, vessel wall injury can result in cytokine release and edema, leading to the collection of inflammatory debris, fibrin, and other molecules, which further exacerbate skin ischemia and leg ulcers [125].

Venous leg ulcers usually occur when the pressure within the veins of the lower extremities rises due to central vascular abnormalities. This persistent high pressure can progressively damage and weaken the small blood vessels in the skin. It often manifests itself on the inside of the leg, between the knee and the ankle, causing discomfort, itching, and swelling in the afflicted leg.

The prevalence of leg ulcers in individuals with SCD is unknown. Further, predicting which individuals are at risk for developing this (and other) complications has remained elusive. However, there have been some known connections to venous incompetence and vasomotor alterations [126,127,128]. Patients with leg ulcers tend to report the onset around the second decade of life and tend to occur ten times more frequently than in the general population [3]. In a study of a large cohort of individuals with SCD conducted by Minniti et al., almost 20% developed ulcers during their lifespan [129]. The most common predictors for leg ulcer development in this cohort were previous trauma, HbSS genotype, male sex, age above 20, and low hemoglobin F (HbF) level [3,130]. An additional study using the Bethesda Cohort, which included over 500 SCD individuals, showed that 22% of those with HbSS and 9% of those with HbSC patients had a history of ulcer [131].

There is little evidence available to guide the management of leg ulcers in SCD. Several RCTs have demonstrated benefit with topical antibiotics [132]. Evidence for using hydroxyurea or CRCT is not clear, with studies supporting each intervention, and no current randomized controlled studies [133,134]. Pharmaceutical therapies such as vascular, antioxidants, growth factors, Hb treatments stimulators, in addition to topical treatments (wound care, antibiotics, growth factors, steroids), and surgical/nonpharmaceutical agents are among the recommended treatments for leg ulcers for those with SCD. Unfortunately, optimal treatment is not yet established.

### 4.6. Avascular Necrosis of Bone

Osteonecrosis, also known as avascular necrosis (AVN), is a condition that occurs when the blood flow to bones is temporarily or permanently interrupted, resulting in cellular death. All cell types in the bone and marrow are affected by AVN (osteocytes, hematopoietic cells, and adipocytes) [135]. AVN is a serious complication of SCD that usually affects the distal portion of long bones (femur, humerus) where there is poor collateral circulation [136].

The exact cause of vascular occlusion leading to AVN is not well defined, but the complication is seen in all genotypes of SCD [4]. AVN may be asymptomatic until late-stage illness, and once symptomatic, there is rapid progression to collapse [137,138]. Currently, there are no optimal treatments identified for either the prevention or treatment of AVN in SCD. Although treatments have been tried, including core decompression, the application of growth factors, and other methods, there have not been any randomized controlled trials performed to determine an optimal method. Without a proven treatment option for AVN, there is currently no reason to screen patients, and the actual prevalence of this condition remains unknown.

In some studies, AVN of the femoral head is associated with risk factors such as frequency of painful episodes, age, alpha-gene deletion, and hemoglobin level. Approximately 50% of patients with HbSS disease have AVN by a mean age of 33 years, while those with HbSS-alpha thalassemia and HbSS-Beta-0 thalassemia are more likely to develop AVN in childhood. AVN is also very common in people with HBSC disease and has been demonstrated in up to 50% of SCD patients by the age of 35 [139].

At present, the current disease-modifying therapies have not been shown to reduce or prevent the development of AVN. There are both non-surgical and surgical options for treating AVN in individuals with SCD, which have not been studied in any randomized trials. Non-surgical approaches include observation, analgesics, physical therapy (to strengthen surrounding muscles), and reducing weight bearing over the joint. Pain management is often needed as well, and many people with SCD require long-term opioid therapy due to the chronic pain. Surgical options include joint replacement, nucleus decompression, bone graft, and osteotomy [140,141]. Unfortunately, this complication results in severe morbidity in people with SCD without significant optimal treatment options.

### 4.7. Sickle Cell Kidney Disease

Kidney complications of SCD include glomerular and tubular pathologies associated with vascular inflammation, endothelial damage, and direct hemolytic damage [142,143]. Over time, the repeated injuries to the glomerulus and tubules lead to the development of albuminuria and decreased glomerular filtration rate. In addition to direct glomerular and tubular injury, the renal vasculature is a generally hypoxic environment made worse by sickle hemoglobin-laden red cells. The inner medulla’s environment (low oxygen tension, hypertonic, and low pH) promotes RBC sickling. It is postulated that severe vascular injury occurs within the vasa recta, leading to its destruction and inability to concentrate urine as early as infancy [144,145,146,147].

People with SCD who develop chronic kidney disease that progresses to end-stage kidney disease/renal failure have high, early mortality and are less likely to receive a renal transplantation [148,149,150]. Therefore, understanding the pathophysiology and natural history of the development of renal failure is key so that interventions can be tested to ameliorate this disease trajectory.

Severe anemia/hemolysis, increased ambulatory blood pressure, and hyperuricemia have been associated with the early development of chronic kidney disease, defined by both albuminuria and changes in glomerular filtration rate (GFR). Sickle cell nephropathy (SCN) [151,152,153,154] may be similar to the diabetic mechanism of renal disease in which patients develop early hyperfiltration followed by a decline in GFR, suggesting these findings can be used as early markers of chronic kidney disease [155]. When the decline in GFR continues into adulthood, those patients with a rapid decline in GFR are at the highest risk for development of end-stage kidney disease and mortality [156,157]. In the most recent evaluation of almost 2000 Black SCD patients who developed end-stage kidney disease (ESKD) in the last 19 years, their mean age was 43 years, which was significantly younger than 58 years for Black patients without SCD [158]. Further, almost 20% of the ESKD cases occurred in younger people with SCD (≤30 years), while only 4% of patients without SCD were <30 years of age.

Another possible risk factor for the increased prevalence of early CKD in SCD is the frequent and repeated development of acute kidney injury (AKI). There is a well-established bidirectional relationship in the general population that exists between CKD and AKI [159]. In adults, those with a history of AKI are more likely to develop progressive CKD [160]. In both pediatric and adult populations with SCD, the incidence of AKI is high during emergency room visits and hospitalizations [160,161], especially those due to vaso-occlusive pain crisis. Data suggest that hemolysis and free heme may be toxic to the renal tubules and promote the development of acute kidney injury [161,162]. Further, studies of patients with SCD during crisis and at steady state have shown elevated markers of tubular and glomerular injury, suggesting subclinical renal injury is also highly prevalent during acute pain events associated with VOC [163,164].

Some strategies exist to prevent or delay the development of CKD; focus should be placed on early interventions prior to the development of higher stages of CKD. Patients receiving hydroxyurea have improved outcomes when started earlier in life and prior to the development of more severe albuminuria [165,166]. Once patients develop albuminuria, the ASH guidelines suggest the use of renoprotective agents, including angiotensin converting enzymes or angiotensin II receptor blockers [167]. Patients who develop ESKD without other significant co-morbidities should be evaluated for a kidney transplantation [168].

### 4.8. Retinal Complications

Sickle cell retinopathy (SCR) is the most serious ocular complication of SCD. SCR is a result of damage to the retina’s microcirculation, which in turn leads to ischemic maculopathy and peripheral occlusions [169]. Retinopathy can be divided into two umbrella groups (non-proliferative and proliferative) characterized by the presence or absence of new, or proliferating, blood vessels.

Goldberg’s [170,171] classification is used to classify and separate the severity of retinopathy into five stages [172]: peripheral arterial occlusion, peripheral arteriovenous anastomoses (dilated pre-existing capillaries also known as hairpin loop), neovascularization/fibrous proliferative often occurring at the posterior border of non-perfusion and appears white, vitreous hemorrhage, and tractional retinal detachment Similar to other retinal vascular illnesses such as retinopathy of prematurity and diabetic retinopathy, these stages are based on clinically significant abnormalities in the retinal vasculature associated with disease development, this method is like. These stages are as follows in order:

Ocular complications among individuals with the HbSS genotype of SCD worsen with increasing age, are more prevalent in adults than children, and affect more males than girls [169,173,174]. However, ocular complications of proliferative sickle retinopathy (PSR) are more characteristically seen in people with HbSC and HbSβ+ disease, rather than in those with HbSS disease. Signs of ocular damage in SCD found in the posterior segment include salmon patches, which initially appear red but over time become salmon in color due to the hemolysis of red blood cells [175]. Other findings in the posterior segment are sunburst lesions, choroidal damage, and vascular tortuosity [176,177].

Current data suggest that people with the HbSC type of SCD have a higher chance of having PSR compared to those with HbSS and HbSβ0 [178,179,180]. The reason for this discrepancy is unknown; however, one potential hypothesis is that people with HbSC disease tend to have higher levels of hematocrit, which could increase blood viscosity, slowing blood flow and causing micro-occlusions in the tiny blood vessels in the retina [181]. Once oxygen extraction is decreased, the angiogenic response in the eye (similar to that in the brain) is the development of new, fragile blood vessels. Advanced therapy for SCR specifically targets the neo-angiogenesis using an intravitreal injection of an anti-vascular endothelial growth factor. This treatment can be both safe and effective, though it has not been well studied in SRC. Research at the molecular level has shown that VEGF-A facilitates vascular permeability and angiogenesis by engaging with VEGF receptor 2 (VEGFR-2) on vascular endothelial cells [182]. This interaction results in the disruption of capillary endothelial tight junctions and the formation of endothelial cell fenestrations, thereby compromising the integrity of blood vessels. Additionally, VEGF-A prompts endothelial cell proliferation and migration, which are hallmark changes in the early stages of angiogenesis [183,184,185]. Current therapies targeting VEGF include pegaptanib [186], ranibizumab [187], and bevacizumab [188].

One technique that can be trialed to prevent or slow the development of SCR is the use of hydroxyurea or CRCT, which has shown to prevent SCR in children [189]. However, currently, there is little evidence on whether hydroxyurea is beneficial over a long period of time in the prevention of SCR. Similarly, anecdotal reports of people with SCD on CRCT appear to be at less risk of developing SCR, but no research has been done to confirm this postulation.

Lastly, in PSR, chronic vaso-occlusion of the retina and the resulting ischemia can prompt the formation of characteristic pathological “sea fan” neovascularization in the peripheral retina [170]. Interestingly, spontaneous auto infarction of sea fans may occur in up to two-thirds of PSR [190], leading to prolonged and severe vision impairment [191,192]. In people in which auto infarction of these blood vessels does not occur, recent studies have demonstrated that scatter laser treatment for sea fans is both effective and safe compared to feeder vessel techniques [193]. Without a randomized controlled trial, however, the optimal approach to treatment of the neovascularization is unknown.

### 4.9. Retinal Artery Occlusion

Another vascular complication of the eye in SCD is central retinal artery occlusion (CRAO). This occurs when the central retinal artery is abruptly blocked, causing reduced blood flow to the retina, rapid cellular deterioration, and vision impairment [194]. The survival of retinal tissue relies on collateral circulation and the duration of ischemia. Immediate diagnosis and early intervention to remove or dissolve the blocking vaso-occlusion (or embolus or thrombus) is essential to prevent permanent retinal harm and vision loss [195].

Several case studies have shown an association between SCD and CRAO [196,197,198,199]. In some cases, erythrocytapheresis has been used acutely, as is done in people with acute ischemic stroke (to rapidly remove sickle-laden RBC and replace them with healthy cells). As a result, many treat CRAO in SCD as an acute stroke requiring urgent intervention to prevent vision loss [199].

### 4.10. Splenic Complications of SCD

Under normal circumstances, as blood circulates through the spleen, plasma is removed to allow macrophages and the reticuloendothelial system to bind pathogens or deformed RBC and remove them from circulation. For individuals with SCD, the removal of plasma increases hematocrit and viscosity and can predispose them to acute splenic sequestration crisis, whereby splenic retention of RBC leads to a rapid decrease in hemoglobin level, hypovolemic shock, and death [200]. Historically, this complication typically occurs within the first decade of life and represents a leading cause of mortality in childhood, particularly for those with HbSS [200,201,202,203]. Along with immediate transfusion of red blood cells, individuals who survive their first episode typically undergo splenectomy and require chronic transfusions given the high rate of recurrence and increased mortality associated with subsequent episodes [201,204,205,206].

However, recent data show more events of splenic sequestration occurring in adulthood, especially in people with heterozygous disease, which needs to be considered by adult-focused physicians caring for at-risk individuals [207,208].

Outside of acute sequestration, people with SCD are at risk for chronic complications of the spleen. Auto infarction of the spleen arises from chronic sickling that leads to hemorrhage, infarction, and ultimately fibrosis [209]. Functional asplenia is characterized by the presence of circulating Howell-Jolly bodies and decreased splenic uptake on scintigraphy, which renders individuals susceptible to infection by pneumococcal and other encapsulated organisms [206,210]. On the other hand, some individuals may develop hypersplenism, a syndrome characterized by splenomegaly with cytopenia(s), reticulocytosis, and circulating Howell-Jolly bodies [200].

To address chronic complications, prophylactic splenectomy in children with SCD reduces the risk of splenic sequestration, transfusion requirements, and splenomegaly without increasing the risk of post-operative infection [211]. However, recent work has shown that children with SCD who initiate hydroxyurea therapy earlier in life may prevent splenic dysfunction and avoid the need to undergo splenectomy [212,213,214,215,216]. Similarly, chronic transfusions may also reverse auto infarction in individuals up to the age of 21 [217,218,219]. Conversely, individuals with HbSC disease are often not afflicted by splenic auto-infarction [208]. In these circumstances, affected persons remain at long-term risk of splenic sequestration or hypersplenism as noted above and may require intervention later in life.

### 4.11. SCD Hepatopathy

An important and often underrecognized manifestation of inflammation-driven organ damage in SCD is hepatopathy. Chronic hemolysis, recurrent vaso-occlusion, and sustained systemic inflammation contribute to hepatic sinusoidal obstruction, ischemia–reperfusion injury, and progressive liver inflammation. Over time, these processes may promote hepatic fibrosis and, in severe cases, progression to cirrhosis. In addition to transfusion-related iron overload and cholestatic injury, emerging evidence highlights a direct role for inflammation and endothelial dysfunction in driving SCD-associated liver pathology. Both clinical and translational studies have highlighted the complex multifactorial nature of sickle hepatopathy encompassing acute hepatic crises, chronic hepatopathy, iron overload, and, in advanced cases, liver failure requiring transplantation [220]. Notably, recent studies using murine models of SCD demonstrate that chronic inflammatory signaling promotes hepatic injury and fibrotic remodeling, providing mechanistic support for inflammation-driven hepatopathy in SCD [63]. Recognition of liver involvement is critical, as SCD-related hepatopathy contributes significantly to morbidity and may be underdiagnosed in clinical practice.

### 4.12. Mortality

As a result of the significant organ complications of SCD resulting in early mortality and the considerably large population of affected individuals, SCD remains a significant public health concern [221,222,223]. While SCD birth rates have generally remained consistent across countries, the actual number of infants born with SCD has increased from 453,000 to 515,000 over the past two decades. Thus, the prevalence of SCD has increased worldwide since the turn of the century [224]. Mortality rates associated with SCD have also risen, resulting in a total of 376,000 deaths, of which 284,000 deaths were thought to be directly caused by SCD in 2021 [225]. Although medical advancements have significantly improved outcomes for individuals with SCD, especially in high-resource countries, mortality rates remain higher than those of the general population [226]. There is an ongoing need for better SCD surveillance [221,227], worldwide newborn screening [221], and improved treatment access, in the United States, but especially in regions like sub-Saharan Africa and South Asia [225].

The quality-adjusted life year (QALY) serves as a broad gauge of disease burden, encompassing both the quality and quantity of life experienced. In terms of quality-adjusted life expectancy and lifetime income compared to individuals without SCD. Individuals with SCD tend to have a significantly lower life expectancy, quality-adjusted life expectancy, and lifetime income. Specifically, life expectancy for those with SCD has been estimated at 54 years, compared to 76 years for those without SCD. Quality-adjusted life expectancy for SCD patients is approximately half that of individuals without SCD. As a result, the reduction in life expectancy is a substantial loss of lifetime income, with individuals with SCD expected to lose approximately $700,000 in lifetime income compared to those without SCD [228].

In addition to low quality-adjusted life expectancy in individuals with SCD, the threat of early mortality remains high, especially for those with severe vascular complications or frequent hospitalizations. Some known risk factors that contribute to early mortality include PH, ESKD, and other comorbidities. The risk of premature death is even higher for those with multiple complications. According to multivariable modeling, iron overload, depression, pulmonary hypertension, and gout are independent predictors of mortality [89,229,230].

The increased risk of premature death in SCD is irrespective of the genotype [89,226,231]. One study, conducted at King’s College Hospital in London, UK, examined a cohort of adult patients from 2004 to 2013 and found that 6.0% of patients died during the study period, with a median age of 42 years at the time of their death. The group with HbSS/HbSβ0 genotype had a significantly lower median survival rate than individuals with the HbSC genotype [226]. However, those with HbSC continued to have a survival rate significantly less than that of the unaffected population.

**Figure 4:**
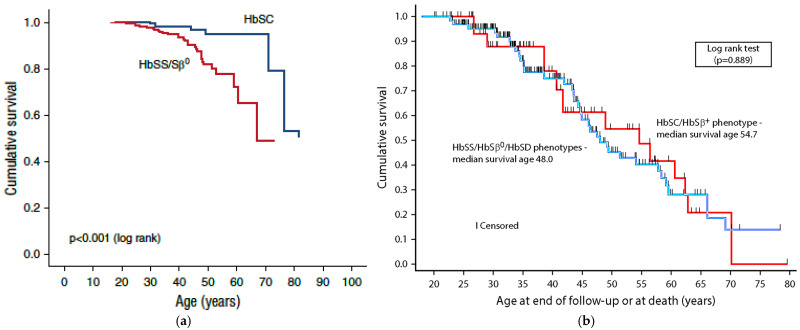
Kaplan-Meier Survival Curves. (**a**) Survival curve by sickle genotype in a UK cohort [226]. (**b**) Survival curve by sickle cell genotype in two SCD centers in the US [231].

In another UK study [231], it was shown that people with SCD had a median survival age of 67 years, which is significantly higher than a recent US cohort study where the median survival age as 44.7 years (Figure 4a). The US study data continued to show a decreased survival in all SCD genotypes, with the median survival age of 48 years for those with HbSS/HbSβ0/HbSD phenotypes and 54.7 years for those with HbSC/HbSB+ phenotypes (Figure 4b). The US analysis accounted for left truncation bias, which means that the estimate of survival was conservative, and the researchers did not investigate extensive risk factors for death [231]. 

### 4.13. Nongenetic Factors of Mortality

As SCD is an inherited condition, it has been primarily studied in terms of genetic variants. However, research has shown that nongenetic factors also play a significant role in explaining clinical variability. Factors such as climate, air quality, socioeconomic conditions, and access to high-resource health care have a direct impact on SCD outcomes. For example, children with SCD living in high-resource countries have better survival rates than those living in sub-Saharan Africa, despite having similar genetic backgrounds.

The effects of climatic factors on SCD are unclear, but some studies suggest that cold weather can cause acute complications [232]. Humidity and wind speed also play a role in pain episodes, with higher levels associated with increased hospital admissions. Air pollution [233], including pollutants like nitric oxide and carbon monoxide, may also have an impact on SCD outcomes, but the relationship is complex and not yet fully understood. In addition to these factors, environmental factors like home conditions [234] and altitude, as well as infectious diseases such as malaria and pneumococcal infections, can contribute to SCD complications and mortality, especially in lower-resource countries. Therefore, understanding and addressing these nongenetic factors are just as essential for improving SCD management and outcomes globally [224].

## 5. Discussion

SCD is a complicated, highly heterogeneous disorder that results in significant morbidity and mortality. While SCD is multifaceted, this review article is focused on the pan-vasculopathy of SCD and the resulting organ complications as demonstrated in Figure 3. While there has been substantial research conducted on the complications of SCD, insufficient funding has been given to establishing a longitudinal clinical registry to better understand disease phenotype and associated outcomes. While certain genetic factors [235] do play a role, there is significant variability in how people experience and respond to the disease that may be based on their access to care and treatment. Environmental influences [232,236], co-existing health conditions [237,238,239,240,241], and socio-economic factors [242,243,244] may also contribute to this variability. As such, it is crucial to gain a more comprehensive understanding of the factors that influence the development of complications to create personalized treatment strategies and improve outcomes. At this time, there are no biomarkers to identify which affected children will develop severe SCD that can be used to optimize management.

Both palliative and transformative treatments are available for SCD, including hydroxyurea, HSCT, and gene therapies [245,246,247]. However, the long-term effectiveness of these treatments in real-world settings remains uncertain. Clinical trials provide some insights, but they often have limited sample sizes and short follow-up periods [248,249]. HSCT studies have effectively demonstrated promising outcomes, but have not captured SCD-organ-specific outcomes. Therefore, it is crucial to have a cooperative, longitudinal registry specific to SCD that includes sociodemographic information, SCD-specific biomarkers and laboratory assessments, and treatment characteristics to assess their relationship with long-term outcomes.

It is essential to provide ongoing specialized care for individuals with SCD, including regular monitoring, preventive assessments, and prompt management of complications [250]. SCD care must include a multidisciplinary approach that encompasses a patient-centered approach, needing both physical and mental health assessments and access to all approved treatments. However, access to such healthcare services can be limited, particularly in underserved and rural communities [251,252]. Improving access to specialized healthcare providers, educating patients and caregivers on the importance of regular follow-up visits, and implementing interventions to address barriers to care, such as transportation issues [244] and financial constraints [253,254], are necessary steps [244].

Importantly, disparities in access to specialized SCD care extend beyond rural and underserved regions in high-income countries to a global scale, particularly in sub-Saharan Africa, where approximately 60% of the world’s population of individuals with SCD resides. Despite experiencing similar end-organ complications, patients with SCD in low-resource settings often have markedly different disease trajectories, characterized by higher childhood mortality and increased morbidity. These differences are driven largely by limited access to early diagnosis, specialized care, disease-modifying therapies, and longitudinal follow-up, rather than by biological differences alone. Advanced therapies such as hematopoietic stem cell transplantation and gene-based treatments remain largely inaccessible in many African settings due to cost, infrastructure requirements, and limited specialist availability. These global disparities further underscore the urgent need for scalable, affordable approaches to SCD care, including improved clinical registries, appropriate biomarkers, and implementation strategies that can inform both high- and low-resource settings.

## 6. Conclusions

This article is designed to provide a thorough review of the SCD-induced vasculopathy to ensure appropriate prevention and assessment can be undertaken for all individuals with SCD. The review is also structured to discuss the use of current SCD-therapies, including several areas in which data are noticeably lacking. There are gaps in knowledge in SCD regarding risk factors for complications, treatment efficacy, and access to specialty care. To address these gaps, a multifaceted approach is required, including establishing longitudinal registries, increasing disease-specific care centers, and ensuring individuals with SCD are referred to specialists [227,255]. Further, the multi-organ pathology induced by SCD requires the team effort of multiple specialties in both research and clinical management. By collectively addressing these challenges, we can advance our understanding of SCD and enhance the quality of care for affected individuals.

## Data Availability

No new data were created or analyzed in this study.
